# First documentation of Rickettsia conorii infection (strain Indian tick typhus) in a Traveler.

**DOI:** 10.3201/eid0705.017527

**Published:** 2001

**Authors:** P Parola, F Fenollar, S Badiaga, P Brouqui, D Raoult


### First Documentation of *Rickettsia conorii* Infection (Strain Indian Tick Typhus) in a Traveler

**To the Editor:** Spotted fever group rickettsiae are gram-negative intracellular bacilli associated with arthropods, mainly ticks, as vectors. To date, 12 tick-borne rickettsioses are recognized worldwide, seven since 1991 (1). Indian tick typhus (ITT) is a tick-borne rickettsiosis prevalent in India (1). Although the disease has been recognized clinically, cases have been documented only rarely, and then mainly with nonspecific serologic tools, such as the Weil Felix test. We report the first serologically documented case of infection caused by *Rickettsia conorii* (strain ITT) in a French traveler returning from India.

In September 1999, a 25-year-old woman living in France was hospitalized with a 4-day history of fever, headache, vertigo, malaise, and disturbance of vision, followed 3 days later by arthromyalgia and a rash. On the day of admission, she had returned from a 1-month stay in India. She reported a number of bites by unidentified arthropods during her trip. Her temperature was 40°C with relative bradycardia (72/minute). Physical signs included pharyngitis, transient epistaxis, bilateral conjunctivitis, and a maculopapular rash including petechiae, mostly on the trunk and lower limbs but also on her palms and the soles of her feet. There was no inoculation eschar.

Clinical laboratory findings included increased alanine aminotransferase (56 UI/L), lactate dehydrogenase (1,008 UI/L), C-reactive protein (130 mg/L), and erythrocyte sedimentation rate (74 mm/hour). The kaolin cephalin time was 43 seconds (control 34 seconds). Hemoglobin was 10.5 g/dL, and the mean corpuscular volume was 99.7 fL. Repeated blood smears, blood cultures, and a stool bacterial culture disclosed no pathogens. Serologic tests, including assays for rickettsioses, were negative.

The patient received 5 days of empirical doxycycline treatment (200 mg/day), intravenously for the first 2 days because of vomiting. She became afebrile 2 days after therapy was begun. Subsequently, all her symptoms resolved. Ten days later, the immunofluorescence assay for antibodies reactive with spotted fever group rickettsiae showed increased levels of immunoglobulin (Ig) M (1:256) and IgG (1:1024) against *R. conorii* Seven and IgM (1:512) and IgG (1:2048) against *R. conorii* ITT. Serologic findings were completed by Western blot performed with acute-phase serum, which showed a band of approximately 135 kDa against *R. conorii* ITT, but not *R. conorii* Seven. Cross-absorption studies were performed with convalescent-phase sera. Sera were absorbed with *R. conorii* Seven and *R. conorii* ITT antigens and then tested by immunofluorescence assay for remaining antibodies reactive to both antigens. When absorption was performed with *R. conorii* ITT antigens, serologic testing was negative for antibodies to both *R. conorii* Seven and *R. conorii* ITT antigens. However, when absorption was done with *R. conorii* Seven antigens, subsequent serologic testing was negative for antibodies to *R. conorii* Seven, but antibodies to *R. conorii* ITT remained (1:100). Thus, Western blotting and cross-absorption strongly supported that the infection was due to *R. conorii* ITT.

Although ITT was clinically described at the beginning of the century, the etiologic agent has never been isolated from patients in India, nor has a case been diagnosed by strain-specific serologic testing. A spotted fever group rickettsia was isolated in 1950 from a brown dog tick, *Rhipicephalus sanguineus*, collected in India (2) and assumed to be the agent causing ITT. It was designated as *Rickettsia conorii*, the agent of Mediterranean spotted fever, which occurs all around the Mediterranean and is transmitted by the same tick species. However, the disease as it appears in India differs from the common description of Mediterranean spotted fever. The rash is frequently purpuric, and an inoculation eschar at the bite site is rarely found, as in this case. The disease as known in India is mild to moderately severe, although our case may be considered severe (1,3,4).

Strain differences within the species *R. conorii* may account for differences in clinical presentation. Although different isolates of *R. conorii* can be distinguished antigenically (5-7), molecular taxonomic methods demonstrated recently that these rickettsiae are closely related and cluster together (8-10). Thus, the species is considered by many as *R. conorii*, including four serovars: *R. conorii* with three type strains, Seven being the one most commonly identified in our laboratory in isolates from France, Portugal, North Africa (D. Raoult, unpub. data), Kenya, and Morocco (apparently a unique isolate); *R. conorii* Indian tick typhus; *R. conorii* Astrakhan, and *R. conorii* Israel.

Immunofluorescence is the reference diagnostic method for associated rickettsioses, but cross-reactivity among related isolates confounds interpretation of serologic tests. Cross-absorption tests, especially in conjunction with Western blot immunoassays, can be used to determine the rickettsia species involved, as reported in this case. The higher sensitivity of Western blots compared with immunofluorescence has been demonstrated previously in our laboratory; it is frequently positive in acute-phase sera when antibodies cannot be detected by immunofluorescence (11). In this case, we used *R. conorii* Seven as the type strain of *R. conorii* because it is most closely related to *R. conorii* ITT phylogenetically (10). Although these techniques are time-consuming and available only in specialized reference laboratories, they provide data of importance that allow a better understanding of the epidemiology of rickettsioses.

**Philippe Parola,*† Florence Fenollar,†**
**Sekene Badiaga,* Philippe Brouqui,*†**
**and Didier Raoult†**

*Service des Maladies Infectieuses et Tropicales, CHU Nord, Marseille, France; and †Unité des Rickettsies, Faculté de Médecine, CNRS UMR 6020, Marseille, France

### References

1. Raoult D, Roux V. Rickettsioses as paradigms of new or emerging infectious diseases. Clin Microbiol Rev 1997;10:694-719.
2. Philip CB, Hughes LE, Rao KNA, Kalra SL. Studies on "Indian tick typhus" and its relation to other human, typhus-like rickettsioses. Arquivos do V Congresso International de Microbiologia, Rio de Janeiro, 17-24 de Augosto 1950. 1958;1:571.
3. Panda GS. Rickettsioses in India. In: Kazar J, Toman R, editors. Rickettsiae and rickettsial diseases. Bratislava: Slovak Academy of Sciences; 1996. p. 106-9.
4. Jayaseelan E, Rajendran SC, Shariff S, Fishbein D, Keystone JS. Cutaneous eruptions in Indian tick typhus. Int J Dermatol 1991;30:790-4.
5. Philip RN, Casper EA, Burgdorfer W, Gerloff RK, Hughes LE, Bell EJ. Serologic typing of rickettsiae of the spotted fever group by microimmunofluorescence. J Immunol 1978;121:1961-8.

6. Goldwasser RA, Steiman Y, Klingberg W, Swartz TA, Klingberg MA. The isolation of strains of rickettsiae of the spotted fever group in Israel and their differentiation from other members of the group by immunofluorescence methods. Scand J Infect Dis 1974;6:53-62.
7. Beati L, Finidori JP, Gilot B, Raoult D. Comparison of serologic typing, sodium dodecyl sulfate-polyacrylamide gel electrophoresis protein analysis, and genetic restriction fragment length polymorphism analysis for identification of rickettsiae: characterization of two new rickettsial strains. J Clin Microbiol 1992;30:1922-30.
8. Roux V, Rydkina E, Eremeeva M, Raoult D. Citrate synthase gene comparison, a new tool for phylogenetic analysis, and its application for the rickettsiae. Int J Syst Bacteriol 1997;47:252-61.
9. Fournier PE, Roux V, Raoult D. Phylogenetic analysis of spotted fever group rickettsiae by study of the outer surface protein rOmpA. Int J Syst Bacteriol 1998;48:839-49.
10. Roux V, Raoult D. Phylogenetic analysis of members of the genus *Rickettsia* using the gene encoding the outer-membrane protein rOmpB. Int J Syst Evol Microbiol 2000;50:1449-55.
11. Lascola B, Raoult D. Laboratory diagnosis of rickettsioses: current approaches to diagnosis of old and new rickettsial diseases. J Clin Microbiol 1997;35:2715-27.

### Multidrug-Resistant *Pseudomonas aeruginosa* Producing PER-1 Extended-Spectrum Serine-ß-Lactamase and VIM-2 Metallo-ß-Lactamase

**To the Editor:** In *Pseudomonas aeruginosa*, secondary beta-lactamases with extended substrate specificity can be responsible for acquired resistance to the most powerful antipseudomonal beta-lactams, such as expanded-spectrum cephalosporins and carbapenems (1). A number of these enzymes have been described, including extended-spectrum serine-beta-lactamases (ESBLs) of groups 2be and 2d (e.g., PER-1 and various OXA-type enzymes) (2,3) and metallo-beta-lactamases of group 3 (e.g., IMP-1 and the recently described VIM-1 and VIM-2 enzymes) (2,4,5). The secondary ESBLs can degrade penicillins, expanded-spectrum cephalosporins, and monobactams (but not carbapenems) and are often susceptible to serine-beta-lactamase inhibitors (1-3). The secondary metallo-beta-lactamases, on the other hand, are notable for their carbapenemase activity and can degrade virtually all beta-lactams except monobactams, while being resistant to the currently available inhibitors (1,2,5,6).

On March 2000, a multidrug-resistant *P. aeruginosa* (isolate VA-182/00) was isolated in pure culture from a bronchial washing of a 58-year-old patient with multiple myeloma. The patient had been admitted 15 days earlier to the Varese University Hospital with a diagnosis of pneumonia and had been treated with ciprofloxacin (0.5 g twice a day) plus piperacillin (2 g three times a day) for 12 days, and then with imipenem/cilastatin (0.5 g three times a day). No cultures of respiratory tract specimens were done earlier in hospitalization. Multiple myeloma had been diagnosed in 1997, and the patient had been treated with multiple cycles of antiproliferative chemotherapy and had received autologous peripheral blood stem cell transplantation. According to clinical records, *P. aeruginosa* had not been isolated previously during this patient's protracted illness. In vitro susceptibility testing showed that the *P. aeruginosa* isolate was resistant to mezlocillin, ceftazidime, cefepime, aztreonam, imipenem, meropenem, gentamicin, tobramycin, netilmicyn (MICs, >128 µg/mL), amikacin (MIC, 64 µg/mL), ciprofloxacin and levofloxacin (MICs, >32 µg/mL). Only piperacillin and piperacillin/tazobactam had MIC values slightly lower than the breakpoints for resistance (64 µg/mL and 48/4 µg/mL, respectively), although–considering the normal MICs of piperacillin for susceptible *P. aeruginosa* (2-8 µg/mL)–it was evident that the isolate also had considerable biological resistance to these drugs. A double disk-diffusion test, carried out with standard disks placed 20 mm apart (center-to-center), showed synergy between clavulanate and aztreonam. The treatment was changed to piperacillin/tazobactam (4 g four times a day), and a slow recovery ensued over a 30-day period. The patient died 3 months later following a relapse of the underlying malignancy.

The unusually high carbapenem MICs exhibited by VA-182/00 suggested production of a secondary metallo-beta-lactamase, while the synergy between clavulanate and aztreonam suggested production of a secondary serine ESBL. A crude extract of that isolate, assayed spectrophotometrically (7), exhibited imipenem-hydrolyzing activity (94 nmol/min/mg protein, inhibited by EDTA) as well as aztreonam-hydrolyzing activity (11 nmol/min/mg protein, resistant to EDTA). Analytic isoelectric focusing (IEF) of the extract, followed by development with the nitrocefin chromogenic substrate (7), showed three bands of beta-lactamase activity of pIs 5.4, 5.6, and 6.3, suggesting the presence of at least three different secondary enzymes. A colony-blot hybridization with probes for the $bla_{IMP}$, $bla_{VIM}$, and $bla_{PER}$ resistance genes (all of which have been previously detected in *P. aeruginosa* clinical isolates from the same hospital [8,9; Luzzaro F, unpub. data]) yielded positive results with both the $bla_{VIM}$ and the $bla_{PER}$ probes. Amplification of the resistance genes by polymerase chain reaction (PCR) with primers VIM/DIA-f (5'-CAgATTgCCgATggTgTTTgg) and VIM/DIA-r (5'-AggTgggC-CATTCAgCCAgA) for $bla_{VIM}$ genes (4,5) and BLAPER-f (5'-gggACA(g/A)TC(g/C)(g/T)ATgAATgTCA) and BLAPER-r (5'-ggg(C/T)(g/C)gCTTAgATAgTgCTgAT) for $bla_{PER}$ genes (9), yielded amplicons of the expected sizes (522 and 966 bp, respectively). Direct amplicon sequencing identified the two beta-lactamase determinants as $bla_{VIM\text{-}2}$ (5) and $bla_{PER\text{-}1}$ (10), respectively, a finding consistent with the pIs 5.6 and 5.4 beta-lactamase bands detected in IEF (3,5). Conjugative transfer of the resistance determinants to *Escherichia coli* proved unsuccessful. In a Southern blot analysis of total undigested DNA from VA-182/00, both the $bla_{VIM}$ and $bla_{PER}$ probes apparently hybridized to the chromosomal DNA band; no plasmid bands recognized by either probe were detected. A PCR experiment with primers OXA10-f (5'-ggAA-CAAAgAgTTCTCTgCC) and OXA105-r (5'-TTAgCCAC-CAATgATgCC(C/T)TC), suitable for amplification of $bla_{OXA}$ genes of the OXA-10 group, did not yield an amplicon of the expected size (719 bp), suggesting that the pI 6.3 beta-lactamase band detected by IEF did not correspond to an enzyme of this group.

This is the first observation of a *P. aeruginosa* clinical isolate simultaneously producing a secondary PER-1 ESBL and a secondary metallo-beta-lactamase. The finding, observed in a hospital where both the resistance genes

